# Endowment effect in capuchin monkeys

**DOI:** 10.1098/rstb.2008.0149

**Published:** 2008-10-06

**Authors:** Venkat Lakshminaryanan, M Keith Chen, Laurie R Santos

**Affiliations:** 1 Department of Psychology, Yale UniversityNew Haven, CT 06520, USA; 2 School of Management & Cowles Foundation, Yale UniversityNew Haven, CT 06520, USA

**Keywords:** capuchin monkey, endowment effect, token exchange

## Abstract

In humans, the capacity for economically rational choice is constrained by a variety of preference biases: humans evaluate gambles relative to arbitrary reference points; weigh losses heavier than equally sized gains; and demand a higher price for owned goods than for equally preferred goods that are not yet owned. To date, however, fewer studies have examined the origins of these biases. Here, we review previous work demonstrating that human economic biases such as loss aversion and reference dependence are shared with an ancestrally related New World primate, the capuchin monkey (*Cebus apella*). We then examine whether capuchins display an endowment effect in a token-trading task. We identified pairs of treats (fruit discs versus cereal chunks) that were equally preferred by each monkey. When given a chance to trade away their owned fruit discs to obtain the equally valued cereal chunks (or vice versa), however, monkeys required a far greater compensation than the equally preferred treat. We show that these effects are not due to transaction costs or timing issues. These data suggest that biased preferences rely on cognitive systems that are more evolutionarily ancient than previously thought—and that common evolutionary ancestry shared by humans and capuchins may account for the occurrence of the endowment effect in both species.

## 1. Introduction

Economists have classically assumed that human decision makers are well-designed rational actors, equipped with neural mechanisms and cognitive strategies that allow them to act in ways that maximize their expected utility. This traditional view of humans as rational strategists, however, comes into conflict with a growing consensus among social scientists that humans consistently behave in ways that are systematically inconsistent with their own rational self-interest. In situations as diverse as judging the quality of cash gambles or deciding between risky and safe alternatives, people regularly violate the tenets of rational choice theory, often basing their preferences on a variety of arbitrary factors that matter little for the decision at hand (Tversky & Kahneman 1981, 1986). A number of classic studies have demonstrated that human decision makers weigh losses more strongly than equally sized gains (Tversky & Kahneman 1981). In addition, when a decision between a safe and risky gain is reframed as a decision between a safe and risky loss, people's preferences shift dramatically from being risk-averse to risk-seeking (Tversky & Kahneman 1986).

Another famous demonstration of our irrational decision-making strategies is the phenomenon termed the endowment effect—the tendency of human decision makers to systematically overvalue objects that they own over objects that they do not yet own. In one of the most famous demonstrations of this effect, Kahneman *et al*. (1990) gave human participants a new object (e.g. a mug) and then offered them the chance to sell this object or trade it for an equally priced alternative good (e.g. a set of pens). Kahneman and colleagues observed that participants consistently refuse to trade their owned object and demanded approximately twice as much money to sell the object as buyers were willing to pay to obtain the object. As this and many other similar studies have demonstrated (Kahneman *et al*. 1991), human decision makers appear to value an object differently after they have become its owner. Since these initial studies, the endowment effect has standardly been observed in a variety of situations, both in the laboratory and in the field (e.g. Thaler 1980; Kahneman *et al*. 1991; Johnson *et al*. 1993; Franciosi *et al*. 1996).

### (a) The origins of the endowment effect and other economic biases

Economists commonly consider the endowment effect and other behavioural biases to be violations of standard rational choice theory. In the case of the endowment effect, for example, rational decision makers should be indifferent between keeping the item they currently have and swapping it for an equally valued item. Nevertheless, real decision-makers' preferences appear to be heavily influenced by ownership in a variety of situations. In addition, the endowment effect (and possibly other behavioural biases, see Santos & Lakshminarayanan 2008) appears to emerge in the absence of much experience. Harbaugh *et al*. (2001) were the first to explore whether children also place a higher value on objects they own over objects they do not yet own. They endowed 6-, 8- and 10-year-old children with a toy and allowed them to trade the toy for an alternative toy that was equal in value. Children showed an endowment effect just as adult participants (e.g. Kahneman *et al*. 1990); they were reluctant to trade an object that they owned for an equally valued alternative. This study and others suggest that behavioural biases such as the endowment of effect can emerge in the absence of much market experience.

The pervasiveness and early emergence of the endowment effect and other biases provide hints that these strategies may be far more basic to human cognition than these biases are often considered. Indeed, the prevalence of at least some of these biases in market-inexperienced children (e.g. Harbaugh *et al*. 2001) suggests that cultural learning and market experience may play relatively little role in the development of these biases. Instead, the early emergence of these behavioural biases hints that more basic cognitive mechanisms might be involved, and that the cognitive architecture giving rise to these mechanisms may be phylogenetically older than previously suspected.

### (b) A new methodological approach: an evolutionary examination of behavioural biases

Our work seeks to address this possibility more directly. To do so, we have begun exploring the evolutionary history of our behavioural biases using a comparative approach. Specifically, over the past few years, we have begun examining the extent to which our human behavioural biases are shared with our closest living evolutionary relatives—the extant non-human primates (e.g. Santos & Lakshminarayanan 2008; Chen *et al*. 2006). Our work has focused on one model species—the brown capuchin monkey (*Cebus apella*), which is a common non-human primate model of human cognition (e.g. Fragaszy *et al*. 2004).

The broad goal of our research was to examine the nature of capuchin monkeys' economic strategies in contexts that were similar to those used in human studies. Unfortunately, most human studies present participants with gambles involving monetary pay-offs, rewards not typically used with non-human subjects. To get around this issue, we developed a form of ‘monetary’ gamble that our capuchin subjects could understand. We (Chen *et al*. 2006) first taught our capuchins that they could exchange small metal tokens with human experimenters for pieces of food. A number of primate species have successfully learned to exchange tokens in this way (e.g. Westergaard *et al*. 1998, 2004; Liv *et al*. 1999; Brosnan & de Waal 2003, 2004), and not surprisingly, our monkeys learned to exchange tokens with relatively minimal training. We then placed our newly trained capuchins into an economic market, one in which the monkeys could choose between different human traders offering different kinds of goods at different prices. At the beginning of each session, each monkey subject began with a small ‘wallet’ of tokens and entered the market where two different experimenters offered different goods at different prices. The experimenters showed the monkey what kind and amount of food they were offering for a single token, and the monkey could then choose to trade with whomever it chose. We could then measure each monkey's preferences in terms of the percentage of tokens they traded with each of the experimenters.

We first used this token-trading set-up to explore whether capuchins behave broadly rationally in this new economic market. To do this, we presented monkeys with a choice between traders who offered two different kinds of food that the monkeys liked equally, e.g. apple slices and grapes. When presented with this choice, our capuchins traders spent about half of their tokens on apples and half on grapes. We then introduced a compensated price shift, basically putting one of the goods, say apples, on sale by providing double the quantity for a single token. Our monkeys bought more of the cheaper food when it went on sale, behaving rationally as a human consumer would to this shift in the prices. We then examined whether the capuchins prefer a trading option that weakly dominates, or more specifically, one that provides the most food overall. We presented the monkeys with a choice between one experimenter who always offered (and gave) one piece of apple, and a second experimenter who always offered two pieces of apple but half the time gave one piece, and half the time gave two. Note that this second trader was a risky choice, but he on average gave one and half pieces of apple which was a better deal than the certain one piece of apple offered by the first experimenter. When faced with this choice, the capuchins preferred to trade with the second experimenter, again choosing the option that allowed them to make the most of their token budget (Chen *et al*. 2006).

These results demonstrate a few important features of our capuchin market. First and most importantly, the capuchins seem to understand the market we have created for them; with little training, our capuchins were able to pick up information about each trader's past behaviour and use that information to make informed choices in the market. Second, our monkeys appear to behave rationally in the market, selectively trading with experimenters who offer them a better deal. Put in more economic terms, our capuchins prefer options that stochastically dominate, ones that tend to give them more food overall. In addition, our capuchins reliably shift their consumption to the cheaper good when the prices change, just as humans do.

Having established that capuchins behave broadly rationally in some aspects of this market, we went on to examine whether capuchins display the heuristics that humans do—namely, reference dependence and loss aversion. In our first study, we presented capuchins with a choice between two traders who gave the same amount of food, either one or two pieces of apple. The first trader, however, gave food by way of a perceived gain. This trader started out by showing the monkey only one piece of apple but when paid gave an additional second piece of apple half the time. The second trader offered the same amount of food by way of a perceived loss. This second trader started out by displaying two pieces of apple but when paid took one of the pieces of apple away half the time. Although the two traders offered the same amount of food on average, our capuchin subjects did not treat them equally. Instead, our monkeys significantly preferred to trade with the experimenter who gave a perceived gain over the one who gave a perceived loss. Interestingly, the monkeys behaved much like human participants in classic behavioural economic studies (e.g. Tversky & Kahneman 1981, 1986)—they evaluate their choices in terms of an arbitrary reference point, namely the initial amount of food that they were shown. We then went on to examine whether capuchins showed this pattern because they were seeking out perceived gains or whether they were instead avoiding perceived losses. Monkeys were given a choice between one trader who always showed one piece of apple and delivered that piece and second experimenter who always showed two pieces of apple but delivered only a single piece. Again, even though both experimenters gave the same pay-off, our capuchins reliably avoided the experimenter who gave less than what he initially offered, suggesting that capuchins, like humans, are averse to losses.

These results suggest that, despite their obedience to rational price theoretic predictions, capuchins appear to exhibit the same systematic behavioural biases that humans display. Capuchins avoided trading with experimenters who gave them perceived losses (i.e. capuchins demonstrated loss aversion) and preferred to trade more with experimenters whose final food offering was more than the initially displayed amount of food (i.e. capuchins demonstrated reference dependence). Capuchin monkeys thus appear to share a number of the systematic biases that humans demonstrate, suggesting the possibility that these biased strategies may have been shared by a common ancestor between humans and capuchins and thus could have emerged over 30 million years ago.

### (c) The present studies: an endowment effect in non-human primates?

Here, we report a new set of studies aimed at investigating whether capuchins share another of humans' irrational tendencies. Specifically, we examine the extent to which this ancestrally related primate species exhibits a bias analogous to the endowment effect. In contrast to the other behavioural biases previously observed in capuchins, there is reason to suspect that the endowment effect is unique to humans. Specifically, social scientists commonly view the endowment effect as resulting from either a concept of ‘ownership’—a sophisticated notion that one can or should hold exclusive control over an object or good (see Kahneman *et al*. 1991; Beggan 1992; Franciosi *et al*. 1996)—or a rich self concept. Considering an endowment effect from this perspective, it is might seem unlikely that non-human primates might share this bias (but see Hauser 2000). By contrast, other researchers have hypothesized that the endowment effect results from a simpler process, perhaps simply as a result of loss aversion (e.g. Kahnehman *et al*. 1990). From this perspective, one might predict that species demonstrating loss aversion would also be likely to exhibit an endowment effect.

A recent report by Brosnan and colleagues provides strong hints that at least one primate species, the chimpanzee (*Pan troglodytes*), exhibits an endowment-like effect in a trading paradigm. Specifically, Brosnan *et al*. (2007) presented chimpanzees with either a piece of food or a toy and then allowed chimpanzees to trade this object for a different but slightly more preferred object. Although most chimpanzees readily exchanged toys for equivalent toys, a reliable number of chimpanzees were reluctant to trade their endowed pieces of food for other slightly preferred foods. In this way, chimpanzees appear to exhibit a behaviour analogous to a human endowment effect, at least when endowed with food as opposed to non-food items.

Brosnan and colleagues have provided the best evidence to date that at least a closely related non-human primate exhibits an endowment effect. Unfortunately, however, their results are open to at least two deflationary alternative explanations. The first interpretation concerns the possibility that chimpanzees' unwillingness to trade their endowed food items may have resulted from the cost of trading the food. It is possible that chimpanzees may be averse to trading rather than eating their endowed food simply because the act of trading involves a certain amount of effort (or what economists might call a transaction cost). Consequently, it is possible that chimpanzees refused to trade with experimenters not owing to an endowment effect *per se*, but instead because they were averse to the additional effort associated with approaching the experimenter and trading the food. A second similar alternative account involves the extra time it takes to trade as opposed to eat the endowed food. Although chimpanzees are known to delay gratification in some circumstances (e.g. Rosati *et al*. 2007), they still may be reluctant to wait for food when another food is immediately available. If chimpanzees were reluctant to wait to obtain the traded food, then the bias towards keeping the endowed food observed in this task may be due more to the delay associated with trading the food than to an endowment effect *per se*.

The goals of our study were threefold. First, we wished to build on the work of Brosnan and colleagues and account for possible deflationary interpretations of these previous findings in chimpanzees. Specifically, we aimed to examine whether a similar endowment effect would emerge when primate subjects were adequately compensated for both timing issues and transaction costs. Second, we wanted to explore the endowment effect in an even more distantly related primate species, one that shared an even more ancient common ancestor with humans. For this reason, we focused on the brown capuchin, which shared a common evolutionary ancestor with humans over 30 million years ago (Fragaszy *et al*. 2004) and is therefore more distantly related to humans than chimpanzees, a species whose common ancestor split from our species' lineage only 6 million years ago (Tomasello 1999). Finally, we wanted to demonstrate the existence of an endowment effect with a primate population that is known to exhibit rational market behaviour at least in some circumstances. Chen *et al*. (2006) previously demonstrated that brown capuchins exhibit very sophisticated, economically rational behaviour in a token exchange market. The capuchins in Chen *et al*.'s study rationally shifted their trading preferences depending on the difference in the quality of the rewards offered by experimenters, obeying standard price theoretic models just as humans do. This population's rational market performance in a trading situation therefore provides a useful benchmark against which to test for an irrational bias such as the endowment effect since this populations' previous performance indicates that they can behave rationally in some market contexts—they rationally respond to price cuts, re-budget tokens towards cheaper goods in a way that is well described by rational maximizer models, etc.

**Figure 1:**
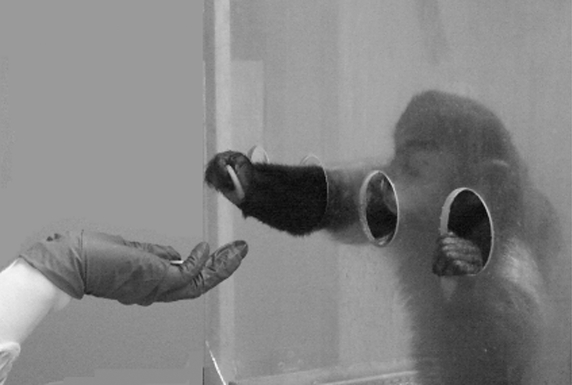
A photograph depicting the token exchange method in capuchins. Here, one capuchin subject, Auric, trades a token for a food reward.

With these goals in mind, we presented the same capuchin monkeys previously tested by Chen *et al*. (2006) with a situation analogous to the experimental markets in which humans demonstrate the endowment effect (e.g. Kahneman *et al*. 1991). Our method, however, capitalizes on the same market task in which this population previously exhibited rational behaviour, a token-trading task (figure 1). We first show that capuchins exhibit an endowment effect in this trading market, and then attempt to rule out three separate alternative explanations for this effect.

## 2. Material and methods

### (a) Subjects

We tested five adult capuchins—two males (NN and FL) and three females (HG, MD and JM), all of whom had previously participated in token-trading experiments (Chen *et al*. 2006). All monkeys had *ad libitum* access to water and were fed a daily food allotment of monkey chow and fruit in the mornings and evenings.

### (b) Apparatus

Subjects were tested in a cubic wire mesh-trading chamber (each side: 83×83 cm) elevated approximately 75 cm and attached to their main home enclosure. The walls on the left and right side of the trading chamber had two openings (5 cm high×8 cm long), such that monkeys could reach through only one side of the box at each time. For each condition, traded objects consisted of either a 2.5 cm metal disc (hereafter, the token) or a food reward. Food rewards included fruit discs (approx. 3 cm in diameter), cereal cubes (1.25 cm cubes of mini-wheat cereal), or a 1.25 cm×0.64 cm slice of marshmallow fluff-filled fruit roll-up (hereafter, FFRU). Experimental sessions were videotaped with a Sony digital-8 videocamera.

### (c) General procedure

Each subject began with a *baseline session*. At the beginning of this session, 12 tokens were placed in the trading chamber. Subjects were then allowed to enter the trading chamber and could use the tokens to purchase rewards by placing a token into the hand of one of the two experimenters (E1 and E2 wearing different colours). To begin each trial, the experimenters positioned themselves on opposite sides of a trading chamber and prepared to trade, leaving one hand open and partially extended into the enclosure to receive a token, and with the other displaying a dish with a food reward within the sight of the subject, but out of its reach. During the baseline session, one experimenter offered a fruit disc while the other offered a chunk of cereal. The monkey was then allowed to choose one experimenter by reaching through the opening with its token and presenting the token into the chosen experimenter's hand. The chosen experimenter moved his dish of food reward within reach of the subject. After each trade, experimenters switched sides, and displayed their offers to begin a new trial. Subjects completed 12 trials per session and thus completed a session once they had spent their entire budget of 12 tokens. If the subject exhibited a preference for one of the two goods (i.e. chose to consume either one of the goods for more than 7 of these 12 trades, indicating a greater-than-chance preference), then we changed the type of fruit and cereal used, and reran preference testing until the subject chose equally across the two presented goods.

After completing the baseline sessions, in which we established the subjects' indifference when choosing to trade tokens for either good, subjects were moved to an experimental session. The experimental session (experiment 1) differed from the baseline sessions in two key ways: first, we replaced subjects' tokens with foods and, second, we presented only one experimenter as a trading option to the subject. Subjects participated in two experimental sessions: one in which they were endowed with fruit discs and could trade with an experimenter offering cereal, and one in which they were endowed with cereal and could trade with an experimenter offering fruit discs. Before running the first experimental session of each condition, subjects each performed one additional *familiarization session* with the new single trader. During these familiarization trials, the single experimenter wore the same colour and delivered the same rewards as in the experimental sessions. In this familiarization session, however, subjects were endowed with four tokens and could trade these with the experimenter who offered one of the two kinds of foods (either cereal or fruit, depending on the condition). This session served to familiarize the subject with the behaviour of this trader.

After this familiarization session, subjects were tested on two experimental sessions in which they were endowed with food instead of tokens. Subjects then had 12 trials in which they were endowed with one kind of food and had a choice between eating the endowed food objects or trading them for the offered equivalent (e.g. endowed fruit disc could be traded for the offered cereal cube, and the endowed cereal cube could be traded for the offered fruit disc). Thus, the subject was allowed to eat as many units of the endowed good as it saw fit, and trade the remainder to the experimenter for the other type of good. Subjects ran one session of 12 trials in which they were endowed with cereal and were offered fruit, and another session of 12 trials in which they were endowed with fruit and offered cereal.

In experiment 2, we examined whether subjects understood that food rewards could be traded. To look at this, we examined whether subjects would trade the endowed cereal or fruit for a more highly valued food item. Experiment 2 used the same experimental set-up as experiment 1, but instead of offering an equivalent good in exchange for the endowed good, the experimenter offered a highly valued treat, an FFRU. Subjects again ran one session of 12 trials in which they were endowed with cereal and offered FFRU, and another session of 12 trials in which they were endowed with fruit and offered FFRU.

Experiment 3 examined whether subjects would continue to show an endowment effect after they had been compensated for the cost of transacting the trade. Experiment 3 presented subjects with a choice between eating their endowed food objects and trading them for the equivalent food plus a small compensation for the transaction cost of the trade. Before running experiment 3, however, each subject was administered a *transaction cost assessment* session to determine the smallest compensation that the subject would accept in exchange for a token. To determine this, each subject was given 12 tokens just as in the baseline session, with only one experimenter available to receive these tokens. For the first round of this transaction cost assessment, the experimenter offered only one piece of uncooked oatmeal (‘1 oat’) in exchange for each of the monkey's tokens. If subjects refused to trade any of their tokens for just 1 oat, then the transaction cost assessment was rerun with the experimenter now offering two pieces of oats in exchange, and so on. In this way, we were able to determine the transaction cost (or minimal compensation necessary for the delivery of the token) for each subject. Experiment 3 then followed the exact procedure of experiment 1, except that the reward offered by the experimenter was increased by the amount of the transaction cost of the trade. Subjects ran one session of 12 trials in which they were endowed with cereal and were offered fruit *plus the oat transaction cost*, and another session of 12 trials in which they were endowed with fruit and offered cereal *plus the oat transaction cost*.

Experiment 4 explored whether subjects' endowment effect was due to temporal discounting problems. In other words, did subjects really exhibit an endowment effect or did they instead choose to keep rather than trade the endowed food simply because it was faster to eat the endowed food than to trade it for the equivalent offered food. To get at this, we presented subjects with a choice between eating an endowed slow-to-eat food object (an almond inside of its shell) or trading it for an equivalent good that was faster to eat (an almond without a shell). Each subject was given 12 slow-to-eat almonds and allowed to trade with one experimenter offering almonds without a shell. Subjects could therefore choose to keep and eat the in-shell almond, and endure the delay associated with opening the nut or exchange it for a more quickly eaten almond with no shell. In this way, we were able to determine whether the capuchins' tendency to keep the endowed good remained even when the endowed good was slower to eat than the offered good. Subjects ran a single session of 12 trials in which they were endowed with in-shell almonds and offered out-of-shell almonds.

## 3. Results

### (a) Baseline

**Figure 2:**
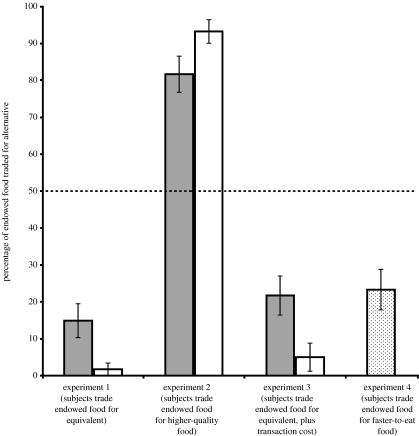
Percentage of endowed food traded by the subject for the alternative food in each condition, pooled across subjects (*n*=60 for each bar). Error bars represent 1 s.e. In experiment 1, when sujects could trade an endowed good for its equivalent, subjects preferred the endowed good over the good available through trade. This preference to consume endowed food, rather than exchange it for an equivalent, persisted despite increasing the size of the offer to account for the cost of the transaction (experiment 3) and the time of the trade (experiment 4). However, subjects were willing to trade food in exchange for a highly valued alternative (experiment 2). Grey bars, cereal; white bars, fruit; dotted bar, in-shell nut.

In the baseline session, one experimenter offered a fruit disc while the other offered a chunk of cereal. Subjects received 12 tokens and could spend each on one of these two food options. Subjects chose equally, spending no more than 7 of their 12-token budget on either of these options. Pooling across all five subjects, monkeys chose cereal exactly as often as fruit, and therefore chose neither option any greater than chance, as confirmed by a binomial probability test (pooled proportion of choices to cereal: 50%, *n*=60, *p*=1.00, see figure 2). This was confirmed with follow-up binomial probability tests for each of our five actors, confirming that no capuchin preferred cereal over fruit in his or her baseline session (proportion of choices to cereal—NN: 50%, *n*=12, *p*=1.00; HG: 58.33%, *n*=12, *p*=0.774; MD: 58.33%, *n*=12, *p*=0.774; FL: 41.66%, *n*=12, *p*=0.774; JM: 41.66%, *n*=12, *p*=0.774). These baseline results confirmed that we had picked two goods of roughly equal value to our subjects (i.e. subjects showed no robust preference between the two goods).

### (b) Experiment 1

Experiment 1 presented subjects with food items in place of their 12 tokens and allowed subjects to trade these food items back to an experimenter who offered an equally preferred food item in exchange. If ownership of these foods does not impact the value that capuchins place on them, then monkeys should consume the same ratio of fruit discs to cereal, as in the baseline. In contrast to this prediction, subjects consumed far more of the fruit discs when they were endowed with fruit discs and far more cereal when endowed with cereal. When endowed with fruit discs, subjects (pooled) spend only 1.7 per cent (*n*=60) of their budget on cereal, and when endowed with cereal, spend only 15 per cent (*n*=60) of their budget on fruit. Both of these percentages are significantly less than 50 per cent (*p*<0.0001, two-tailed binomial tests). All five subjects show this same pattern individually. For all five subjects, we can reject the null hypothesis that they prefer cereal and fruit equally regardless of endowment; for all subjects (individually), this is rejected in a two-sample test of equality of proportions (Fisher's exact probability test) at the 0.1 per cent level for four of our five subjects and at the 2 per cent level for the fifth (HG).

### (c) Experiment 2

To ensure that this pattern of results does not reflect subjects' unwillingness to use food as tokens, experiment 2 presented subjects with sessions in which they were allowed to trade their endowed good for a treat of far greater value, the FFRU. We observed that subjects had no trouble exchanging food for this higher good, and ate significantly less of the endowed quantities when the FFRU was available in exchange. Subjects traded away significantly greater than half of their endowed fruit discs or cereal for FFRU, trading 93.3 per cent (*n*=60) and 81.67 per cent (*n*=60) of the time, respectively. Both of these percentages are significantly more than 50 per cent (*p*<0.0001, two-tailed binomial tests). All five subjects show this same pattern individually, trading significantly more than 50 per cent of the time for FFRU. This is significant for all five subjects at the 5 per cent level in a two-tailed binomial test when they were endowed with fruit and for four of our five subjects when they were endowed with cereal.

### (d) Experiment 3

Experiment 3 then addressed a second alternative for the endowment effect exhibited in experiment 1: monkeys may be reluctant to trade their endowed food due to the cost of transporting the endowed food to the experimenter for exchange. To address this transaction cost alternative, we first estimated the transaction cost of trading. Endowing subjects with tokens as in the baseline test, we gradually increased the exchange value until subjects chose to trade rather than keep their tokens. We observed that all subjects were willing to trade a token for a single oat. This quantity of oats (*n*=1), then, served as an estimate of the ‘transaction cost’—that is, the compensation it would take to induce a capuchin subject to engage in the trade. We then reran exactly our earlier two endowment conditions, but offered subjects not only equivalent goods in exchange for their endowed goods, but also offer them an oat in compensation for the effort of completing the trade. We replicated the initially reported pattern of results and found that subjects still prefer more of the endowed good, even if they are compensated for the effort of trading.

Just as in experiment 1, subjects (pooled) traded less than 50 per cent of their budget of endowed food for an equally attractive food (plus a single oat). When endowed with fruit discs, monkeys traded only 5 per cent of the time (*n*=60) and, when endowed with cereal, traded only 21.7 per cent of the time (*n*=60). Both of these percentages are significantly less than 50 per cent (*p*<0.0001, two-tailed binomial test). This pattern holds across all subjects; all five subjects traded less than 50 per cent of their endowed foods when they could exchange these for the equally attractive food (plus one oat). This is significant for all five subjects at the 5 per cent level in a two-tailed binomial test.

### (e) Experiment 4

Finally, experiment 4 explored another alternative, namely that subjects exhibit an endowment effect in experiment 1 simply because trading takes more time than eating the food. To get at this, we presented subjects with the option to trade a slow-to-eat almond inside its shell for a more quickly eaten almond outside its shell. Just as in experiments 1 and 3, subjects traded less than half of their endowment for an option that took less time to eat, in this case, an out-of-shell almond. Given a total of 60 in-shell almonds, which could either be eaten or traded for out-of-shell almonds, subjects (pooled) traded only 23.33 per cent of the time. This is significantly less than 50 per cent (*p*<0.0001, *n*=60, two-tailed binomial test).

These results were confirmed by follow-up tests conducted for each subject. Four of our five subjects traded less than 50 per cent of their endowed in-shell almonds when they could exchange these for more quickly eaten out-of-shell almonds. NN traded 0 per cent of his endowment of in-shell almonds (*n*=12). HG also traded 0 per cent of her endowment (*n*=12). FL and MD each traded 8.3 per cent of their endowments (*n*=12 for each monkey). These four subjects each trade significantly less than 50 per cent of the time for the more quickly eaten option (*p*<0.01, two-tailed binomial tests). An additional monkey, JM, showed the opposite pattern, trading 100 per cent of her endowed in-shell almonds (*n*=12).

## 4. Discussion

Like humans (Kahneman *et al*. 1990, 1991), our capuchin participants are reluctant to trade food that they own for equally preferred foods. When tested in experiment 1, capuchins preferred to eat fruit discs when they were made owners of fruit discs, but preferred to eat cereal pieces when they were made owners of these objects instead. Monkeys' willingness to sell an item appears to be less than their willingness to buy an identical item. As with previous effects reported in chimpanzees (Brosnan *et al*. 2007), the endowment effect we observe cannot be merely due to an inability to trade food rewards—monkeys tested in experiment 2, readily traded their endowed food object when offered a more valuable food item in exchange. In contrast to previous work in chimpanzees, however, our results cannot be explained with deflationary alternative accounts involving transaction costs and timing effects. Our capuchins subjects continue to exhibit an endowment effect in experiments 3 and 4, even when they are compensated for the time and cost of the transaction. Taken together with Brosnan and colleagues' similar findings in chimpanzees, our results suggest that the bias to overvalue owned objects is not unique to humans. Indeed, this bias appears to be shared with a species who shared a common ancestor with humans over 30 million years ago.

The present results fit with a growing body of work suggesting that many of our own species behavioural biases—reference dependence (Chen *et al*. 2006), loss aversion (Santos & Lakshminarayanan 2008) and now the endowment effect—appear to be shared with other primate species, even those that are distantly related in evolutionary time. Such findings suggest that at least some behavioural biases may not emerge as a result of specific economic experiences and market disciplining—instead, our human systematic biases might be the result of evolved cognitive strategies, ones present in our primate lineage for considerable phylogenetic time. Our work further provides some constraints on the cognitive and neural mechanisms that may underlie these biases in the human species. Specifically, our observation that non-linguistic species share human behavioural biases suggests that these heuristics cannot rely on language or linguistic processing. In addition, our findings hint that such biases cannot be due to complex or uniquely human cognitive capacities, such as a rich sense of self or an explicit notion of ownership.

The possibility that the endowment effect and other behavioural biases result from evolved cognitive strategies raises the question of why these strategies evolved in the first place, and what they might ultimately be for. Economists typically consider behavioural biases such as the endowment effect and loss aversion to be irrational, namely they involve choices and preference reversals that would not be predicted by the tenets of standard utility maximization. The presence of these biases in distantly related primates, however, suggests that such strategies have existed for some evolutionary time, raising the possibility that they might serve some ultimate use (e.g. Gigerenzer & Todd 1999 and Gigerenzer & Selton 2001 for a similar logic about the evolutionary usefulness of purportedly irrational strategies). Our results suggest that researchers should further investigate how and in what circumstances the endowment effect could be evolutionarily useful (see Beggan 1992 and Santos & Lakshminarayanan 2008 for a similar discussion).

Finally, the growing body of work demonstrating the endowment effect in distantly related primates highlights an opportunity for a more rigorous study of the neural basis of these biases. Currently, much work in human neuroscience has begun exploring the neural underpinnings of loss aversion, the endowment effect and other related behavioural biases (de Martino *et al*. 2006; Tom *et al*. 2007). Much of the work performed to date, however, has used functional imaging techniques. Although such techniques have already provided tremendous insight into the neural basis of loss aversion and related biases, such techniques are often the most insightful when used in conjunction with animal-based physiological techniques. To date, little work has addressed the endowment effect and other behavioural biases from a neurophysiological perspective in large part because it was unclear that these biases could be observed in a primate behavioural model. Our work suggests that one could easily develop a monkey model of the endowment effect and thus could develop a primate model for examining the nature of ownership and value at the neural level. The present study therefore paves the way for a neurophysiological investigation of the endowment effect, with the possibility of studying the effect of ownership on value at the level of single neurons. We thus hope that the present work adds both to our understanding of the evolutionary nature of the endowment effect and related biases, as well as to the future of our knowledge of the neural basis of these phenomena.
